# Editorial: Understanding and Engineering Antibody-Superantigen Interactions

**DOI:** 10.3389/fimmu.2022.857339

**Published:** 2022-02-09

**Authors:** Samuel Ken-En Gan, Jeremy P. Derrick, Franca Fraternali

**Affiliations:** ^1^Antibody & Product Development (APD) Lab, Agency for Science, Technology, and Research (A*STAR), Singapore, Singapore; ^2^APD SKEG Pte Ltd, Singapore, Singapore; ^3^James Cook University, Singapore, Singapore; ^4^School of Biological Sciences, Faculty of Biology, Medicine, and Health, University of Manchester, Manchester, United Kingdom; ^5^Randall Division of Cell and Molecular Biophysics, King’s College London, London, United Kingdom; ^6^The Francis Crick Institute, London, United Kingdom; ^7^The Thomas Young Centre for Theory and Simulation of Materials, London, United Kingdom

**Keywords:** superantigens, antibody engineering, protein-protein interactions, antibodies, protein A, protein G, protein L

Superantigens are classically defined as antigens that induce hyperactivation of the immune system. They are traditionally associated with non-specific activation of T-cells that induces abnormal cytokine release. A search for the term ‘superantigens’ on Google in January 2022 yielded results with a heavy bias towards T-cell superantigens (1–4) which were first described in 1989 (5). Almost 30 years later, the T-cell centric view of superantigens continues to overshadow B-cell or antibody superantigens, even though reports of B-cell superantigens date back at least to 1966 for Protein A (6) and 1984 for Protein G (7). Implementation for antibody purification followed rapidly, assisting the development of therapeutic antibodies. In the early 1990s, applications of superantigens expanded towards potential therapeutics, particularly against cancer (8–11). These earlier studies are now bearing fruition: for example, a recent review of the literature on the application of Staphylococcal enterotoxins in cancer immunotherapy found over 50 studies, of which Staphylococcal enterotoxin A was the most common superantigen employed (12). Fusions of superantigens with, for example, antibody Fab fragments which are targeted against tumour-associated antigens have been shown to be effective against melanoma and other cancers (12). Superantigens have also found recent application in CAR-T therapy; von Scheidt et al. demonstrated that inhibition of solid tumour growth in mice was potentiated by pre-incubation of CAR T cells with Staphylococcal enterotoxin B (13). Although most of these studies to date have used mouse models, translation of some applications to the clinic in the near future seems likely.

Intensive research into SARS-CoV-2, driven by the current pandemic, has identified an interesting link between toxic shock syndrome, bacterial superantigens and the viral S, or spike, protein that is the basis for many vaccination strategies against the virus. A sequence motif within the S protein resembles a section within Staphylococcal enterotoxin B. Cheng et al. showed how this can be exploited through development of a monoclonal antibody against the site, which is able to block viral entry into cells (14). This work represents a novel strategy for exploiting a superantigen structure for therapeutic applications.

Given these widespread applications in antibody labelling, purification and immunotherapy, it is apposite to consider what unifying features there may be which apply to the large and diverse superantigen family. A review in this Research Topic uses a broad definition of the term ‘superantigen’ to include antibody-directed, as well as T-cell-directed molecules (Deacy et al.). A number of similarities are identified in the recognition properties between immune macromolecule (e.g. antibody, MHC) and cognate superantigen. Compared with all protein-protein complexes within the Protein Data Bank, such complexes fall within a relatively small range when considering the physical characteristics of each interface. Interestingly, B-cell and T-cell directed superantigens do not obviously segregate (see Figure 9 in Deacy et al.). This observation suggests a degree of structural, as well as functional, congruence which argues for a broader definition of the term ‘superantigen’.

Extending the term superantigen further is likely to prove controversial but we propose that other, probably more remote, additions to the family are likely to be advocated in the future. In this Research Topic, we have one such provocative article (Su et al.) demonstrating superantigen-like binding of nickel to antibodies. Although nickel does not fit the typical definitions of antigen or superantigen, it is a known aggravator of metal allergies. Some investigators have sought to exclude nickel as a formal T-cell superantigen based on the recognition properties (15) alone, despite there being functional similarities by nickel to known superantigens (16).

With recent suggestions that unnecessary immune system exposure to the environment may underlie many disease pathogenesis in the ‘epithelial barrier hypothesis’ (17), there is certainly much to investigate on the roles of superantigens in pathogenesis. It is interesting that, together with superantigens, the ‘epithelial barrier hypothesis’ has intersections with the ‘hygiene hypothesis’, first proposed in 1989 (18); discussions on the intersection are ongoing (19–21). In addition to potential roles in disease pathogenesis implied by both the ‘hygiene’ and ‘epithelial barrier’ hypotheses, superantigen-antibody interactions also underpin the behavior of the natural gut flora by maintaining healthy IgA levels (22). Such interactions may explain the bias in VH genes that mediate Staphylococcal Protein A interaction in humans by Radke et al. Other recent studies have extended such considerations to IgE (23) and, as mentioned above, with respect to nickel binding (Su et al.).

Given the possible confounding interactions from the local microflora on therapeutics in pharmacomicrobiomics, further investigation into superantigen recognition and its biological implications is well justified. A holistic (24) approach, previously used to study the impact of antibody regions (25–30) distally on each other as allosteric, was also used in the Research Topic to find additional target sites in Staphylococcal enterotoxin B (SEB) with therapeutic potential (Bai et al.).

We are presenting in this Research Topic findings that encourage a revision of more restrictive definitions of the term ‘superantigen’ and their applications in biomedicine. We hope this will stimulate novel research approaches that further stimulate a re-examination of the biological functions and applications in therapeutics, diagnostics, and biotechnology of superantigens.

## Author Contributions

All authors contributed to the article and approved the submitted version.

## Conflict of Interest

Author SG was employed by company APD SKEG Pte Ltd.

The remaining authors declare that the research was conducted in the absence of any commercial or financial relationships that could be construed as a potential conflict of interest.

## Publisher’s Note

All claims expressed in this article are solely those of the authors and do not necessarily represent those of their affiliated organizations, or those of the publisher, the editors and the reviewers. Any product that may be evaluated in this article, or claim that may be made by its manufacturer, is not guaranteed or endorsed by the publisher.
